# Electric Charge Effect on the Water Mass Transfer across Mixed Aqueous–Organic Droplet Interfaces

**DOI:** 10.1021/acs.jpca.5c06517

**Published:** 2025-10-30

**Authors:** Mercede Azizbaig Mohajer, Michael J. Gleichweit, Grégory David, Loren Ban, Felix Graber, Ruth Signorell

**Affiliations:** Department of Chemistry and Applied Biosciences, Laboratory of Physical Chemistry, 27219 ETH Zurich, Vladimir-Prelog-Weg 2, Zurich 8093, Switzerland

## Abstract

Understanding gas-particle mass transport is essential for predicting aerosol behavior in the atmosphere and in industrial processes. The mass accommodation coefficient, α_M_, is a key parameter describing this exchange. Defined as the probability of a gas-phase molecule adhering to a particle upon collision, α_M_ presents a highly surface-sensitive property. Aqueous atmospheric aerosol droplets carry electric charges, which accumulate near the surface; however, their influence on the gas-particle mass transport remains elusive. To access the influence of charge on α_M_, we combined an aerosol charging method with photothermal single-particle spectroscopy, enabling direct water mass exchange measurements on the surface of single aerosol droplets. We investigated charged and neutral aqueous glycerol and tetraethylene glycol droplets across a wide range of concentrations and temperatures. The micrometre-sized droplets carried approximately 10^3^ elementary charges—exceeding typical atmospheric aerosol charge levels—yet our results show that α_M_ is independent of the droplet charge and instead is dominated by composition and temperature. Theoretical estimates of the charge-dipole and dipole–dipole interaction energies corroborate this finding, highlighting that under atmospherically relevant conditions, electric charge plays a negligible role in the mass accommodation process.

## Graphical abstract

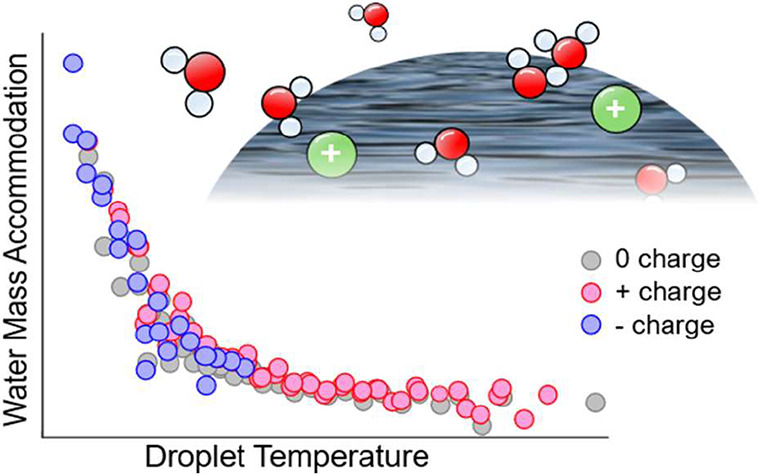

## 1 Introduction

Aerosols, dispersions of small liquid or solid particles in air, play a critical role in industrial processes, the radiative budget of the Earth’s atmosphere and human health.^1^ Atmospheric aerosol particles originate from a variety of natural and anthropogenic sources, or form directly in the atmosphere through nucleation.^2^ These particles span sizes from a few nanometres to tens of micrometres and their high surface-to-volume ratios cause diverse unique physical and chemical processes which affect their properties.^3^ These processes include the heterogeneous uptake of various gases by atmospheric aerosol particles.^4–8^ Understanding the mass transport between gas and condensed phase (i.e., particulate phase) is important across a number of disciplines, from drug delivery systems to cloud microphysics. An important parameter that is used to describe the mass transport of gases through an interface is the mass accommodation coefficient α_M_. Originally introduced by Maxwell,^9^ this coefficient describes the probability that an impinging gas-phase molecule will stick to the surface upon collision. α_M_ influences the rates of condensational growth which is important for cloud formation and climate modeling or the growth of pharmaceutical aerosols in the respiratory tract. However, to the best of our knowledge, no study has yet investigated the potential influence of an important particle property, the net electrical charge, on the mass accommodation of water.

In the atmosphere, aerosol particles and cloud droplets often carry net electrical charges – typically ranging from tens to hundreds of elementary charges.^10–13^ There are many factors that could charge the aerosol particles, including cosmic rays, radiation from radioactive materials, lightning, electromagnetic radiation, high-temperature discharge, and triboelectric effects from particle collisions.^14,15^ When aerosol particles carry electric charges, particle interactions and surface chemical reactions can be altered,^16,17^ potentially changing some physical characteristics such as cohesion, adhesion, and atmospheric stability.^14^ Droplet charge also influences cloud microphysics through several mechanisms. A small fraction of charged droplets can enhance collision rates between droplets and hence initiate raindrop formation.^18,19^ Electrostatic forces significantly increase the probability of aerosol-droplet collisions, with only few tens of elementary charges altering the interaction rates.^13,20^ Additionally, electrical charge can lower the minimum supersaturation required for haze droplets to activate and grow into cloud droplets.^21,22^ Recent studies have reported altered chemical reactivity in charged microdroplets, often attributed to interfacial electric fields or charge-induced molecular orientation.^23–26^ However, the underlying mechanisms driving microdroplet chemistry differ fundamentally from those governing mass accommodation, and therefore charge effects observed in chemical reactivity cannot be generalized to nonreactive uptake processes. Despite the ubiquity of charged droplets in the atmosphere and the effects they have on chemical and physical processes, the influence of particle charge on many other important aerosol processes, such as gas-particle mass transport, remains unknown.

Herein, we combined a controlled aerosol charging technique^27,28^ with our recently developed Photothermal Single-Particle Spectroscopy (PSPS)^29–32^ to quantify water mass transport across charged droplet surfaces. We report the mass transfer of water on charged aqueous tetraethylene glycol (TEG) and glycerol (Gly) droplets over a wide range of concentrations and temperatures. This approach enables us to probe potential charge related effects such as charge-dipole interactions or electrostatic shielding, particularly relevant for polar gas-phase species like water. We found that under atmospheric conditions, the mass transport across mixed aqueous–organic interfaces remains unaffected by the presence of surface charge. This observation has important implications for models of water uptake and evaporation, suggesting that charge effects can be neglected.

## 2 Experimental Section

### 2.1 Droplet Generation, Electric Charging, and Charge Characterization

We generated ensembles of mixed aqueous organic droplets using a medical nebulizer (Pari, Pari Boy SX). The droplets in this study consisted of either aqueous glycerol (Gly) or aqueous tetraethylene glycol (TEG). Both Gly and TEG are fully miscible with water and exhibit no specific surface activity under the studied conditions. Therefore, the surface composition of the droplets is expected to closely mirror their bulk composition. We generated droplets with three different charge states which were controlled in the following ways: First, uncharged droplets were obtained by removing all charged droplets from the nebulized ensemble using an electrostatic precipitator (Cambustion Aerosol Precipitator) operated at a voltage of 2 kV. Charged droplets of both polarities were lost by deposition on the precipitator walls. Second, we generated positively charged droplets by passing the droplet ensemble through a home-built positive corona-wire aerosol charger. Third, we replaced the positive aerosol charger by a negative corona-wire charger, with which negatively charged droplets were obtained (see SI for setup).

To characterize the size and charge of the aerosol droplet ensemble formed by the nebulizer (referred to here as “initial aerosol”), we used a scanning mobility particle sizer spectrometer (SMPS, TSI model 3938), which combines electric mobility sizing with single particle counting.^27^ The size distribution for aqueous TEG droplets directly after nebulization is shown by the blue trace in Figure 1. The distribution spans droplet diameter from approximately 50–600 nm, with two maxima at ∼70 and ∼200 nm. Uncharged droplets cannot be detected by the SMPS under these conditions. These size distributions were determined by using the SMPS equipped with an X-ray neutralizer (TSI model 3088) and by applying the multiple charge correction as implemented in the SMPS software. Notably, the size distribution changes upon charging. To analyze positively charged droplets, the SMPS was operated in negative polarity without the X-ray neutralizer. Conversely, negatively charged droplets were analyzed using positive polarity on the SMPS. The original size distribution shifts substantially to smaller mobility diameter (i.e., higher electrical mobilities), with new maxima centered at ∼20 and ∼65 nm for both charge states. These shifts indicate that the droplets passing through the corona-wire chargers acquire a higher number of charges than those passed through the X-ray neutralizer, as illustrated by the yellow trace for positively charged droplets and the green trace for negatively charged ones in Figure 1. Careful evaluation of these shifts in size distribution revealed average charge states of *q*_initial_ = 2 ± 1, 8 ± 4, and 19 ± 8 elementary charges for initial droplet diameter of *D*_initial_ = 100, 200, and 400 nm, respectively, for both positively and negatively charged aerosols. The uncertainties are estimated from the standard deviation of the mobility distribution to the charged distribution.

**Figure 1:**
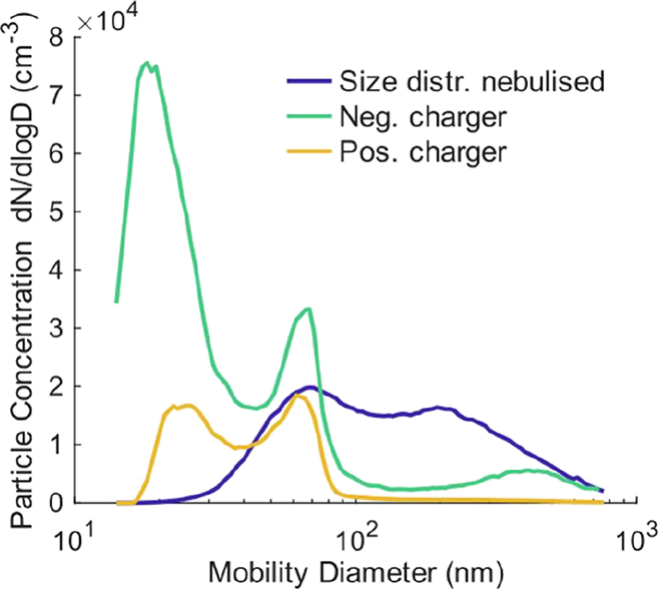
Mobility diameter distributions of nebulized aqueous TEG droplets. The blue trace corresponds to the size distribution for the nebulized aerosol ensemble obtained by equipping the SMPS with the X-ray neutralizer and applying a multiple-charge correction. The green and yellow traces indicate the shift in electrical mobilities by charging the droplets with the negative and positive corona-wire chargers, respectively.

The charged or neutral aerosol ensembles were then directed to the optical trap, where their mass transfer properties were studied using PSPS (described in Sections 2.2 and 2.3). Due to the optical forces acting in the trapping region, the initial aerosols undergo coagulation, forming larger, micrometre-sized droplets that were the focus of this study. Based on the particle size and charge distributions obtained from the SMPS analysis, we performed a Monte Carlo simulation^28^ to estimate the charge state of the resulting TEG droplet in the optical trap. These droplets typically exhibited radii between 1000 and 1500 nm, with corresponding charge states ranging from approximately ±1500 to ±5500 elementary charges (e).

Analogously, the charge states of aqueous Gly droplets were estimated. For droplet radii between 1000 and 1500 nm, we found charge states between 2500 and 8000 e and −1500 and −4500 e for positively charged and negatively charged droplets, respectively (see SI for details).

### 2.2 Optical Trapping

The charged and uncharged aqueous tetraethylene glycol (TEG) and aqueous glycerol (Gly) droplets described in Section 2.1. were immobilized in a humidified nitrogen atmosphere by counter-propagating optical tweezers (CPT). The CPT setup employs a continuous wave trapping laser (532 nm, Laser Quantum, Opus, 400 to 1000 mW), which was protected from back-reflections by an optical isolator (Thorlabs IO-5-532-HP). The laser beam passed through an electro-optical modulator (EOM, Conoptics 350-50-01), was expanded to 7.4 mm in diameter using a beam expander (Edmund Optics #37-053), and then split into two beams of equal intensity using a half-wave plate (Thorlabs, WPH10M-532) and a polarizing beamsplitter cube (Thorlabs, CCM1-PBS251). The Gaussian beams, with orthogonal polarizations, were directed onto two aspherical lenses (75 mm focal length, Thorlabs, ASL10142), which focus the beams into the center of a custom-built photoacoustic trapping cell (PA cell).^33^ The CPT configuration enables narrow particle confinement, while maintaining enough space for optical access and additional measurement probes.

To ensure a tight confinement, stable trapping, and high reproducibility throughout the measurement campaign, we implemented a feedback system. The particle’s position was continuously monitored by detecting elastically scattered light projected onto a position sensitive detector (PSD, lateral effect sensor, Thorlabs PDP90A). The signal from the PSD was processed by a proportional-integral-derivative (PID) controller, which regulated the voltage applied to the EOM. By rotating the linear polarization of the incident laser beam, the EOM adjusted the power in each trapping arm, thereby stabilizing the droplet position in real time. A schematic of the full optical setup is provided in the SI.

### 2.3 Modulated Mie Scattering and In Situ Sizing

The droplet radius *r* and the mass accommodation coefficient α_M_ are both retrieved from the elastically scattered light of the trapping laser by the droplet, based on the method described in our previous work.^29,31,32^ The scattered light is collected over an angular range of 49.0° around a scattering angle of 90° using a microscope objective (Mitutoyo, M Plan Apo 20x). The collected light was then divided using beam splitter cubes and directed onto three detectors: a photodiode (PD, Hamamatsu S2506-02) for determining droplet size and α_M_, a CMOS camera for visual observation of the droplet in the optical trap, and a PSD for feedback stabilization (see Figure 2).

**Figure 2:**
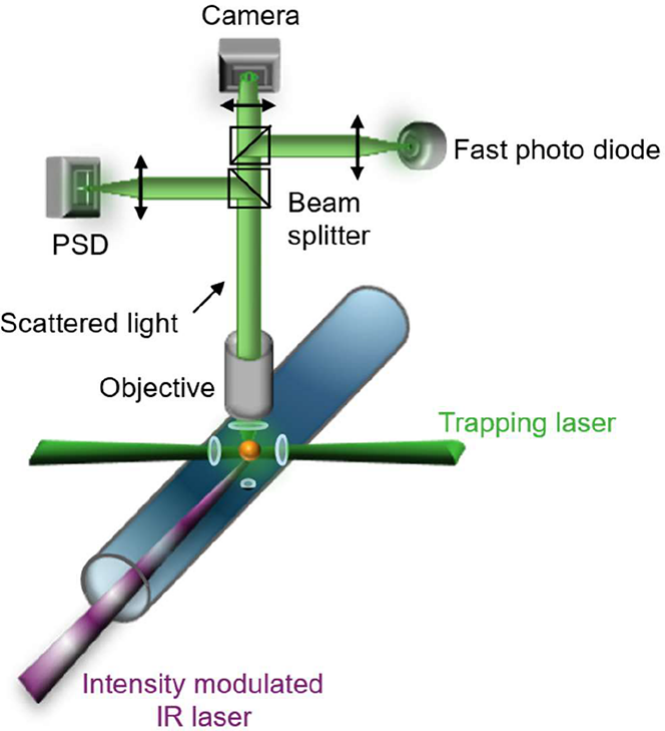
Schematic of the optical setup, showing the trapping laser after the lenses (dark green), a trapped droplet (yellow dot), and the intensity modulated IR laser (purple). The elastically scattered light from the particle was collected by an objective and subsequently split onto three detectors: fast photodiode, CMOS camera, and position sensitive detector (PSD).

To retrieve the mass accommodation coefficient α_M_ (defined in the Section 2.4), we induce a small periodic perturbation in the droplet by an intensity modulated infrared (IR) laser (AdTech Optics), focused precisely onto the trapping position. Two slightly different setups were used for measuring PSPS of Gly and TEG, with the respective setup-specific details summarized in Table S1 of the Supporting Information. The modulated IR absorption leads to periodic changes in droplet temperature, water concentration (and therefore refractive index), and radius. These cyclic variations drive energy exchange between the droplet and the surrounding gas via mass flux *I* (effective evaporation/condensation of water) and heat flux *Q* (causes heat expansion/contraction). The resulting miniscule changes in refractive index and radius manifested themselves as periodic changes in the light scattering pattern. The integral of this scattering pattern over the collection angle was recorded by the PD. By demodulating the PD signal at the modulation frequency using a lock-in amplifier (MFLI, Zurich Instruments, 500 kHz) we extract the Modulated Mie Scattering (MMS) signal. MMS has an amplitude component MMS_A_^exp^, which reflects changes in the particle size and refractive index and a phase component (MMS_Φ_^exp^), which arises from the time delay between the IR laser emission and the scattered light detection. The MMS phase was not used in the following, but only the MMS amplitude.

The particle size was accessed in situ by considering the (slowly changing) DC component of the PD signal, referred to as the Total Two-dimensional Angular Optical Scattering (TTAOS).^34^ Likewise to MMS, TTAOS also depends on droplet properties, such as size and refractive index, and exhibits characteristic Mie resonances at specific particle sizes. Aqueous TEG and Gly slowly evaporate over minutes to hours, which is negligible compared to the modulation period of the IR excitation laser (∼250 μs). The gradual shrinking of the droplet allows us to access a broad range of particle sizes – from 2.0 to 0.4 μm in radius – in a single experiment.

We conducted measurements at various initial droplet sizes and across a wide range of relative humidities (RH), from 13 to 91%. The RH, and thereby the exact droplet composition,^35^ was controlled by a constant flow of humidified nitrogen gas through the PA cell (total flow: 20–30 sccm).

### 2.4 Multilayer Heat and Mass Transfer Model

To derive the mass accommodation coefficient α_M_, the experimentally measured MMS amplitude MMS_A_^exp^ was compared to the simulated amplitude MMS_A_^sim^.^32^ The simulation was performed by using our previously developed multilayer heat and mass transfer model (MHM-PA), which describes the photothermal response of single aqueous droplets exposed to modulated IR laser excitation (Figure 3).^32,36^

**Figure 3:**
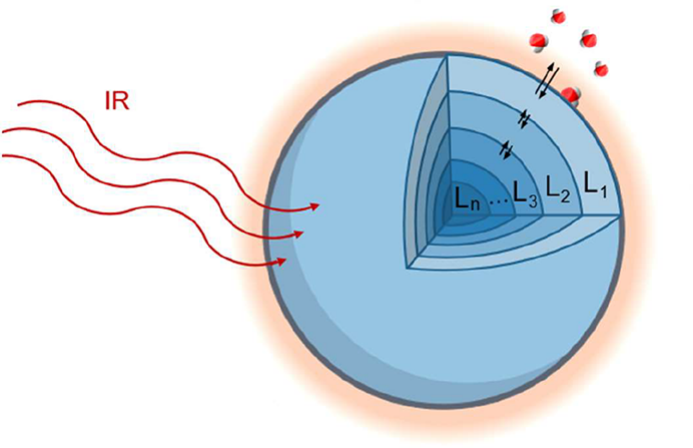
Schematic of the MHM-PA model. The droplet is divided into radial layers of 50 nm thickness. The model calculates the external heat and water mass exchange, as well as internal diffusion of heat and water.

This model combines Mie theory for light scattering with Fourier’s law of thermal conduction and Fick’s first law of diffusion to describe heat and mass flux at the droplet surface. The external heat flux Δ*Q* and water mass flux Δ*I* are given by eqs 1 and 2, respectively.

$$\Delta Q=4\pi r\beta_{T}K(\overset{®}{T})\Delta T$$ 1

$$\Delta I=4\pi r\beta_{M}\frac{D_{M}p_{V}(\overset{®}{T})}{R}\left[\frac{LM_{V}}{R\overset{®}{T}}-1\right]\frac{\Delta T}{(T+\Delta T{)}^{2}}$$ 2

Here, *r* is the particle radius, β_T_ and β_M_ are the transition regime correction factor for heat transfer and mass transfer, respectively, $K(\overset{®}{T})$ is the heat conductivity of the surrounding gas, $\overset{®}{T}$ is the average particle temperature, Δ*T* is the maximum change in temperature at the droplet surface from $\overset{®}{T}$ during a photothermal cycle, *D*_M_ is the diffusion coefficient, *M*_V_ is the molar mass of water, $p_{V}(\overset{®}{T})$ is the water vapor pressure, *L* is the latent heat, and *R* is the universal gas constant. Based on the original work of Fuchs and Sutugin,^37,38^ the transition regime correction factors β_*i*_ for heat and mass transfer (*i = T, M*) can be derived from the Knudsen numbers Kn_*i*_ and expressed by eq 3. The Knudsen numbers are defined as Kn_T_ = λ_N_2__ *r* ^–1^ and Kn_M_ = λ_V_ *r* ^–1^, which depend on the mean free path of the surrounding gas λ_N_2__molecules and water vapor molecules λ_V_, respectively.

$$\beta_{i}=\frac{1+{\mathrm{Kn}}_{i}}{1+\left(\frac{4}{3\alpha_{i}}+0.377\right){\mathrm{Kn}}_{i}+\frac{4}{3\alpha_{i}}{{\mathrm{Kn}}_{i}}^{2}}$$ 3

Here, α_T_ is the thermal accommodation coefficient and α_M_ is the mass accommodation coefficient. Consistent with our previous work and other authors, we set α_T_ = 0.97.^32,36,39^ Importantly, the model does not assume any particular accommodation mechanism, as it relies solely on the net outcome of heat and mass flux. This allows α_M_ to be retrieved without requiring any knowledge on surface charge, electric fields, molecular orientation, or specific molecular interactions at the droplet interface.^32^ The model also does not assume a quasi-static surface or sorption layer, allowing for a small temperature difference between the droplet surface and the surrounding gas.^30^

Within the droplet, small gradients in temperature and water concentration establish, which are described by Fick’s first law of diffusion:

$$\vec{F}=D\vec{\nabla }C$$ 4

where $\vec{F}$ is the heat or mass flux vector, *D* is the heat or mass diffusion coefficient, and *C* is either the temperature or concentration. The model divides the droplet into radial layers, each with time-dependent temperature, water concentration, and volume (see Figure 3). The flux *F* between adjacent layers is described as

$$F_{i,i\pm 1}(t)=k(T,x_{\mathrm{mol}})\delta C_{w,i,i\pm 1}(t)$$ 5

where *k* is the transport velocity for heat or mass, dependent on *T* and the water mole fraction *x*_mol_. For water-TEG and water-Gly simulations, we used a time step of 2 ns and a layer thickness of 50 nm to ensure numerical convergence.

The MHM-PA simulation provides the temporal evolution of *r*, *T*, refractive index *n*, and *C*_*w*_ of each layer of the droplet.^32^ From these quantities, we reconstructed the time-dependent scattering intensity TTAOS­(*t*) for all experimental radii and relative humidities. The MMS amplitude was then derived from the simulated TTAOS­(*t*), described by

$${\mathrm{MMS}}_{A}^{\mathrm{sim},\mathrm{norm}}(\overset{®}{r})=\frac{\left|\max(\mathrm{TTAOS}(t))-\min(\mathrm{TTAOS}(t))\right|}{2\sqrt{2\overset{®}{\mathrm{TTAOS}}}}$$ 6

The normalized simulated MMS amplitude was directly compared to the normalized experimental MMS amplitude to retrieve α_M_. This was achieved by fitting the simulated MMS amplitude to the experimental trace and minimizing the normalized sum of squared residuals. This modeling framework enables precise retrieval of α_M_ under near-equilibrium conditions and captures the dynamic interplay of heat and mass transport within the droplet during IR excitation.

## 3 Results and Discussion

We recorded MMS signal responses for a statistically robust data set, comprising 187 aqueous TEG droplets and 139 aqueous Gly droplets, each studied under three charge states: uncharged, positively charged, and negatively charged. The experiments spanned water mole fractions from 0.12 to 0.85 and average droplet temperatures between 21 and 26 °C. These conditions were experimentally achieved by probing droplets with radii between 0.4 and 2.0 μm, exposed to RHs ranging from 13 to 91% for TEG droplets and 12 to 89% for Gly droplets.

This section is structured as follows: We begin by comparing the MMS spectra of TEG and Gly droplets across the three studied charge conditions. For TEG droplets, we then present the retrieved α_M_ values for charged and uncharged cases. In contrast, α_M_ could not be extracted for Gly droplets, which is further explained in the SI. Therefore, these are discussed based solely on their raw MMS data. Finally, we interpret our results in the context of electrostatic forces present under the experimental conditions.

### 3.1 Charge Effect on α_M_ at 3 Different RH for pos., neg., and Uncharged TEG and Gly

Figure 4 shows the experimentally measured Modulated Mie scattering (MMS) amplitude as a function of droplet radius for aqueous TEG (a–c) and aqueous Gly (d–f) at three RH conditions: 25% (a) and d), 50% (b and e), and 85% (c and f). Each trace represents an average over multiple measurements under identical conditions, and the shaded regions indicate the standard deviation, which reflects the reproducibility of the signals across experiments. For each RH, the MMS amplitude is plotted for droplets that are uncharged (black trace), positively charged (red trace), and negatively charged (blue trace), with charges on the order of 10^3^ elementary charges. Across all conditions, the traces exhibit the characteristic double-peak structure of the MMS signal, which reflects the interplay of refractive index and radius modulation during IR excitation. The behavior of the MMS signal thus serves as a direct measure of the magnitude of water mass flux – and thereby α_M_ – under a given experimental condition.

**Figure 4:**
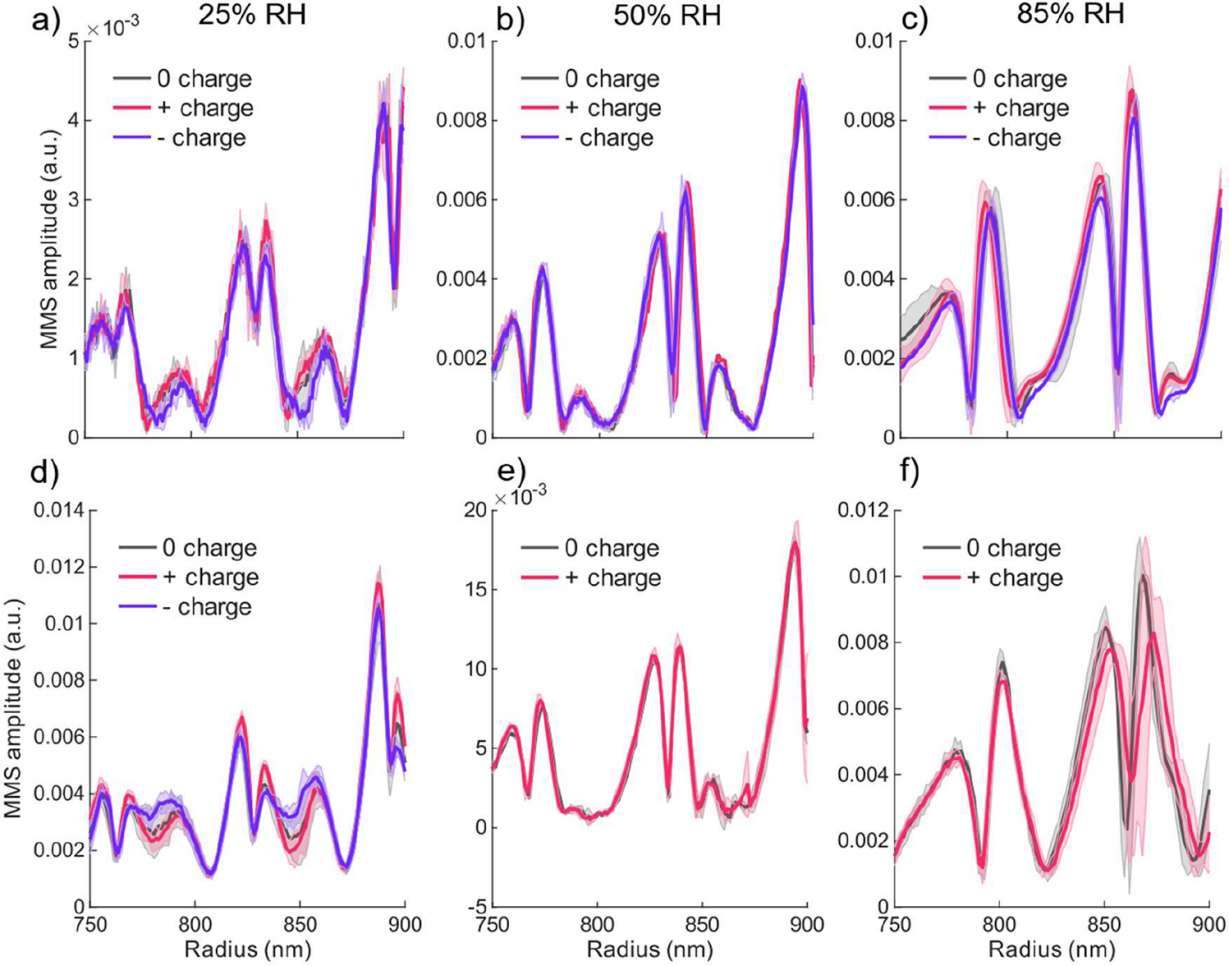
Experimental MMS amplitudes as a function of droplet radius for uncharged (black trace), positively (red trace) and negatively charged (blue trace) aqueous TEG (a–c) and aqueous Gly (d–f) droplets, displayed for three RHs: (a, d) 25%, (b, d) 50%, and (c, f) 85%.

For aqueous TEG droplets, the MMS traces corresponding to all three charge states overlap within the experimental standard deviation (Figure 4a–c), indicating no systematic differences in the water mass flux attributable to the droplet charge. Similarly, for aqueous Gly droplets, we compared MMS traces measured for charged and uncharged droplets. At 25% RH – where positively, negatively charged and uncharged droplets were measured – no differences outside the experimental standard deviations between the traces were observed (Figure 4d). At 50 and 85% RH, only positively charged and uncharged droplets were measured (Figure 4e,f). Again, no observable differences were found. In these cases, only positively charged droplets were analyzed because the estimated number of elementary charges was higher than for negatively charged droplets (see SI).

The above-described comparison of the raw data already suggests that, within the experimental resolution, the photothermal response captured by MMS is not significantly influenced by the net charge of the droplets. Nonetheless, the qualitative differences between the averaged data could hint to miniscule trends in α_M_. Therefore, we studied the effect of electric charge on the water mass transfer more carefully by retrieving absolute values for the mass accommodation coefficient α_M_ of water on charged vs uncharged aqueous TEG droplets.

### 3.2 Mass Accommodation Coefficient of Water on TEG

Figure 5 shows the retrieved mass accommodation coefficient α_M_ of aqueous TEG droplets as a function of water mole fraction *x*_mol, water_ and the average droplet temperature $\overset{®}{T}$ , for three different charge states: uncharged (a), positively charged (b), and negatively charged (c). We observe a complex dependence of α_M_ on the droplet temperature and composition.

**Figure 5:**
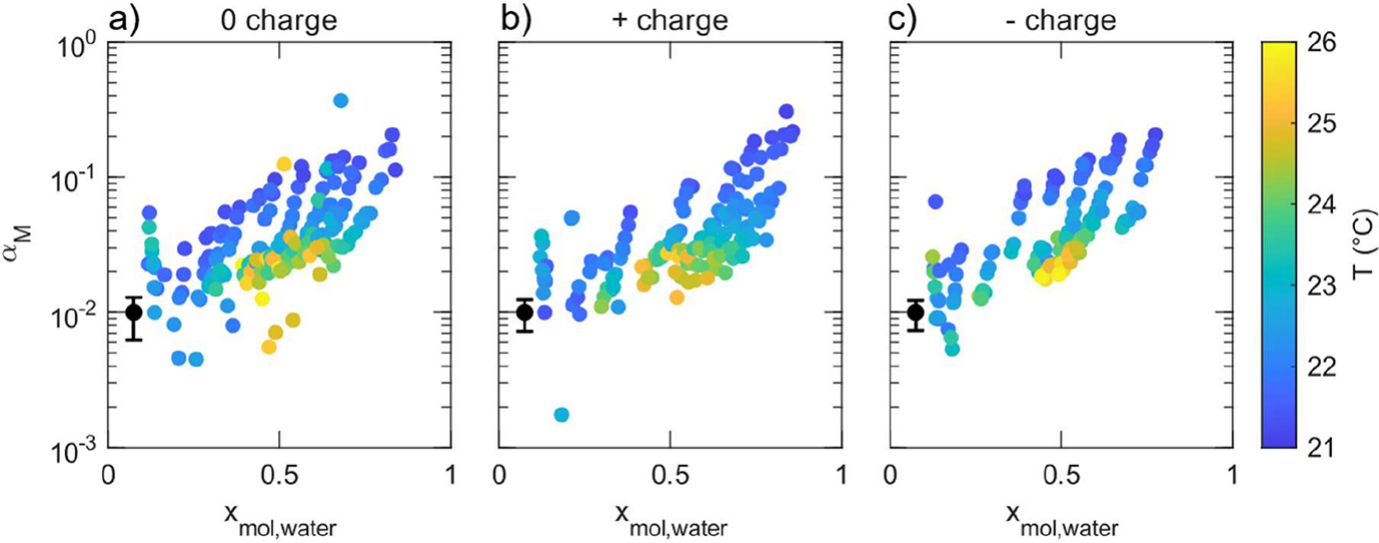
Mass accommodation coefficient α_M_ as a function of the water mole fraction *x*_mol, water_ and droplet temperature (color code) for uncharged (a), positively charged (b), and negatively charged (c) aqueous TEG droplets. The black marker in the lower left corner of each panel indicates the mean fitting uncertainty in α_M_ across all MMS peaks. The uncertainty in the water mole fraction is typically below 0.006 and always below 0.02.

At low water mole fractions *x*_mol, water_ < 0.5, α_M_ remains approximately constant around 0.02 and shows no significant dependence on the water content inside the droplet. This behavior can be explained by the surface composition, which mirrors the droplet’s bulk composition as TEG and water are fully miscible. In our previous work,^30^ we have rationalized that in this regime, most collisions between gas-phase water molecules and the droplet surface occur with TEG molecules due to its greater interfacial presence compared to water. Since water–TEG interactions are less favorable for accommodation than water–water interactions, the probability of successful accommodation remains low and relatively insensitive to further changes in water content. Moreover, within the studied temperature range (21–26 °C), α_M_ does not exhibit a measurable dependence on the droplet temperature.

As the water mole fraction increases beyond 0.5, α_M_ begins to rise and exhibits a pronounced temperature dependence. Our data shows values of α_M_ up to ∼0.2 at the highest measured water content (*x*_mol, water_ ≈ 0.85) and lowest temperature ( $\overset{®}{T}=21$ °C). This increase is attributed to the growing probability of gas-phase water molecules colliding with liquid water molecules at the droplet surface, which facilitates accommodation due to stronger hydrogen bonding and more favorable solvation. The extent of this increase diminishes with rising temperature, and for $\overset{®}{T}>24$ °C, α_M_ stabilizes around 0.02, independent of the droplet composition. The temperature dependence of α_M_ is consistent with an Arrhenius-type behavior, originally described by Davidovits et al.^40^ through transition state theory, and subsequently employed in our previous studies.^29,32^ The accommodation process was treated as a competition between desorption and solvation of surface adsorbed water molecules. At higher temperatures, increased thermal energy favors desorption, which reduces the probability of successful accommodation.

These observations are consistent with previous studies of α_M_ on aqueous TEG droplets.^29–31^ Compared to our earlier work, the present study offers a more advanced and robust analysis by employing the newly developed MHM model.^36^ In addition, we observe clear similarities between TEG and triethylene glycol (TREG), where both systems show decreasing α_M_ with increasing temperature.^32^ However, the concentration dependence in TREG was more complex with a pronounced minimum in α_M_ at intermediate water mole fractions. This was attributed to a saturated hydrogen-bond network between TREG and water at these concentrations. Beyond this minimum, α_M_ increases with increasing water content, analogous to the behavior observed in aqueous TEG droplets.^32^

Importantly, no systematic differences were observed between the three charge states in Figure 5. To facilitate clearer comparison across charge conditions, an overlaid version of Figure 5 with error bars is provided in the SI. The trends of α_M_ with droplet composition and temperature for uncharged (a), positively charged (b), and negatively charged (c) droplets lie within the experimental uncertainty across the entire range of conditions. We further highlight this observation by displaying α_M_ as a function of droplet temperature and a constant water mole fraction of 0.65 ± 0.05 for all three charge states in Figure 6. We again see the clear decrease in α_M_ with increasing droplet temperatures from ∼0.2 to ∼0.02, as the temperature increases from 21 to 24 °C. This trend is consistent across all three charge states, and the data do not indicate any significant or systematic differences between uncharged, positively charged, and negatively charged droplets.

**Figure 6:**
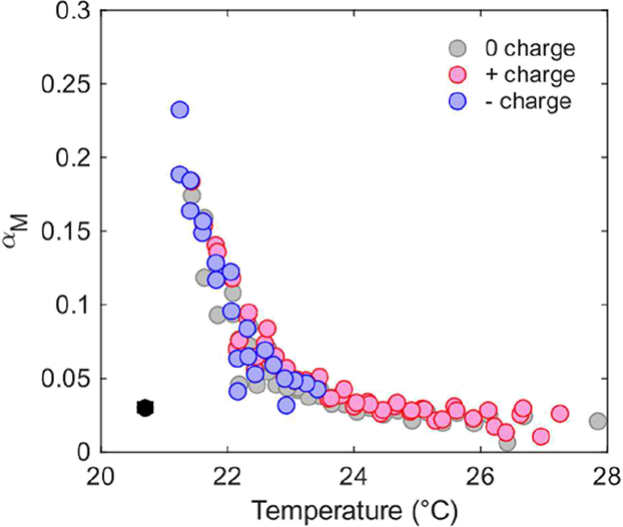
Mass accommodation coefficient α_M_ as a function of droplet temperature for a water mole fraction of *x*_mol, water_ ≈ 0.65. The data are displayed for uncharged (gray dots), positively charged (red dots), and negatively charged (blue dots) aqueous TEG droplets. The black marker in the lower left corner indicates the mean fitting uncertainty in α_M_ across all MMS peaks for the three charge states. The uncertainty in temperature is typically below 0.1 °C and always below 0.25 °C.

### 3.3 Discussion of the Missing Charge Effect

Our measurements show no discernible effect of charge state on the mass accommodation coefficient α_M_. This observation can be rationalized by considering the distribution of charges in conductive liquids. In aqueous droplets, charges predominantly accumulate at the surface due to electrostatic forces. Assuming a water droplet radius of 1 μm and a surface region thickness of 0.2 nm, a droplet carrying 1500 elementary charges contains approximately one charge per 55,830 surface molecules. At such low charge densities, the probability of an incoming water molecule impinging close to a localized charge situated in the surface region is negligible compared to collisions with the neutral surface area between the charges.

The electric field strength generated by 1500 elementary charges at the droplet surface is insufficient to influence the accommodation process. To illustrate this, we estimated the electric field using a simplified model in which the net charge is assumed to be concentrated at the center of the droplet, thereby treating it as a point charge. For a droplet with radius 1 μm and net charge of 1500 e, the established electric field strength at the surface is approximately *E* = 2.2 · 10^6^V/m. The potential energy of a water molecule with dipole moment $\vec{\mu }$ in this field is given by^41^

$$U_{q\mu }=-\vec{\mu }\cdot \vec{E}$$ 7

Comparing this to the thermal energy at 298.15 K yields $\frac{U_{q\mu }}{k_{B}T}\approx 3.3\cdot 10^{-3}$ , which is far below the threshold required for dipole alignment. To achieve noticeable alignment, the electric field energy of the dipole would need to be comparable to or larger than the thermal energy, i.e. the field strength would need to exceed 6.4 · 10^8^V/m. This would correspond to an unrealistically high droplet charge of ∼1.8·10^6^elementary charges – well above the Rayleigh limit of ∼1.3·10^5^ e for a 1 μm droplet. Therefore, under the conditions studied, electrostatic interactions are too weak to influence the accommodation process.

This conclusion is further supported by comparing the interaction energies of charge-dipole and dipole–dipole pairs. The angle-averaged charge-dipole interaction energy *V*_*q*μ_ is described as

$$V_{q\mu }=-\frac{1}{3}\frac{q^{2}\mu^{2}}{(4\pi \varepsilon_{0}\varepsilon_{r}{)}^{2}{r_{q\mu }}^{4}k_{B}T}$$ 8

where *q* is the droplet charge, μ the dipole moment of water, ε_0_ and ε_*r*_ are the vacuum and relative permittivity, respectively, *r*_*q*μ_ is the distance between charge and dipole, and *k*_B_ *T* is the thermal energy. The angle-averaged dipole–dipole interaction energy *V*_μμ_ is expressed as

$$V_{\mu \mu }=-\frac{2{\mu_{1}}^{2}{\mu_{2}}^{2}}{3{\left(4\pi \varepsilon_{0}\varepsilon_{r}\right)}^{2}{r_{\mu \mu }}^{6}k_{B}T}$$ 9

Here, *r*_μμ_ is the distance between two dipoles. While charge-dipole interactions scale with ${r_{q\mu }}^{-4}$ and are theoretically stronger than dipole–dipole interactions, which scale with ${r_{\mu \mu }}^{-6}$ , the effective interaction strength is diminished by the sparse distribution of charges. Assuming a dipole–dipole separation of 0.3 nm (typical molecular distance in water)^42^ and an average dipole-charge separation of approximately 33 nm (see SI for details), the ratio of the respective interaction energies, integrated over a representative surface aera, was computed using a Monte Carlo model (described in the SI), yielding $\frac{V_{q\mu }}{V_{\mu \mu }}\approx 0.06$ . This result indicates that dipole–dipole interactions dominate under these conditions, explaining the observed charge independence of α_M_.

## 4 Conclusions

This study provides the first direct comparison of photothermal single particle measurements of aqueous TEG and Gly droplets across different charge states – uncharged, positively charged, and negatively charged – spanning a wide range of relative humidities, droplet sizes, and temperatures. Uncharged droplets were produced by passing the aerosol ensemble through an electrostatic precipitator, while charged droplets of either polarity were generated using corona-wire chargers. The resulting micrometre-sized droplets acquired about ±10^3^ elementary charges. By leveraging Modulated Mie Scattering and our recently developed MHM-PA model,^32^ we delivered the first quantitative retrievals of the mass accommodation coefficient α_M_ for water on aqueous TEG under controlled charge conditions.

Our results confirm a strong dependence of α_M_ on droplet composition and temperature.^29–32^ At low water mole fractions (*x*_mol, water_ < 0.5), α_M_ remains low (∼0.02) and temperature-independent, consistent with a surface dominated by TEG molecules and limited hydrogen bonding. As the water content increases, α_M_ rises, reaching values up to ∼0.2 at high water mole fractions and low temperatures. This enhancement is attributed to increased H_2_O­(g)–H_2_O­(l) interactions at the droplet surface, which promote accommodation. For *x*_mol, water_ > 0.5, α_M_ becomes strongly temperature-dependent and decreases with increasing temperature. This behavior follows an Arrhenius-type trend, where elevated thermal energy favors desorption over solvation, thereby reducing α_M_.^29^ Notably, we find that typical droplet charges, while being atmospherically ubiquitous, have no significant effect on α_M_. This charge independence is supported by theoretical estimates of the charge–dipole and dipole–dipole interaction energies, which show that charge densities on the droplet surface are too low, and thus electrostatic forces are too weak to influence the accommodation process under the studied conditions. Individual water molecules may impinge near localized charge sites and experience strong electrostatic interaction energies; however, these events are statistically rare due to the low surface charge density and thus do not contribute to the average α_M_.

By systematically isolating and studying the role of droplet charge, we demonstrate that under typical atmospheric conditions, charge effects are negligible. The micrometre-sized droplets in our experiments carried approximately 10^3^ elementary charges, which is higher than the charge levels typically observed in atmospheric aerosol particles (10^1^–10^2^ e). The mass accommodation of water on mixed aqueous–organic droplets is therefore primarily governed by their compositions and temperatures, rather than by droplet charge. This insight has implications for modeling water uptake and evaporation in charged aerosol systems, suggesting that charge effects can be safely neglected under these conditions.

## Supplementary Material



## Notes

Droplet generation, electric charging, and charge characterization, complete optical setup, list of physical constants used in modeling, problems with glycerol multilayer heat and mass transfer simulations, charge independence of the mass accommodation coefficient, and surface charge distribution and electrostatic interactions (PDF)
