# Supplementary material for: Electric Charge Effect on the Water Mass Transfer across Mixed Aqueous–Organic Droplet Interfaces
Source: J Phys Chem A. 2025 Oct 30;129(45):10550–8. doi: 10.1021/acs.jpca.5c06517 (PMC12621247; doi:10.1021/acs.jpca.5c06517)
Supplement: Supplementary file 1 [file jp5c06517_si_001.pdf]

Electric Charge Effect on the Water Mass Transfer Across Mixed Aqueous-Organic Droplet Interfaces

Mercede Azizbaig Mohajer, Michael J. Gleichweit, Grégory David, Loren Ban, Felix Graber, and Ruth Signorell**

Department of Chemistry and Applied Biosciences, Laboratory of Physical Chemistry, ETH Zurich; Vladimir-Prelog-Weg 2, 8093 Zurich, Switzerland

*Co-corresponding authors. Emails: mercede.azizbaig@phys.chem.ethz.ch, rsignorell@ethz.ch

Supplementary Information

1. <u>Droplet Generation, Electric Charging, and Charge Characterisation</u>

Aqueous tetraethylene glycol (TEG) and glycerol (Gly) droplets are nebulised using N_2 gas with 1.5 bar pressure, as shown in Fig. S1. The aerosol ensemble is passed through an electrostatic precipitator and a home-built unipolar corono-wire aerosol charger (with either positive or negative polarity, generating droplets with the respective charge state) with a flow rate of 0.3 lpm. The excess aerosols are removed by an exhaust. With a Scanning Mobility Particle Sizer (SMPS), the size and charge distributions are determined. The Gly droplet size distribution after nebulising is obtained by equipping the SMPS with an X-ray neutraliser and applying the multiple charge correction, and is shown by the blue trace in Fig. S2. Analogously to Fig. 1 in the main, the blue trace represent the distribution obtained after passing the aerosols through the inactive aerosol chargers. Activation of the chargers, resulted in a measurable shift in mobility diameter: the green trace in Fig. S2 corresponds to negatively charged droplets, while the yellow trace represents positively charged droplets. These shifts were used to infer the the number of elementary charges per droplet forming the basis for a Monte-Carlo simulation that estimates droplet charge following coagulation in the optical trap.[1]

As shown in Fig. S2, positively charged droplets exhibit a higher shift in mobility diameter than negatively charged ones, indicating a higher net charge. Based on these distributions, the Monte-Carlo model predicts that Gly droplets with radii between 1000 and 1500 nm carry approximately 2500 to 8000 positive charges and -1500 to -4500 negative charges. The model provides a probability density function for the number of charges a droplet is expected to carry, based on the number of coagulation steps needed to reach the input size range, as shown in Fig. S3 for aqueous

Gly droplets. Fig. S3 left and right show the probability density function for positively and negatively charged Gly droplets, respectively, for a target radius range of 1000 to 1500 nm.

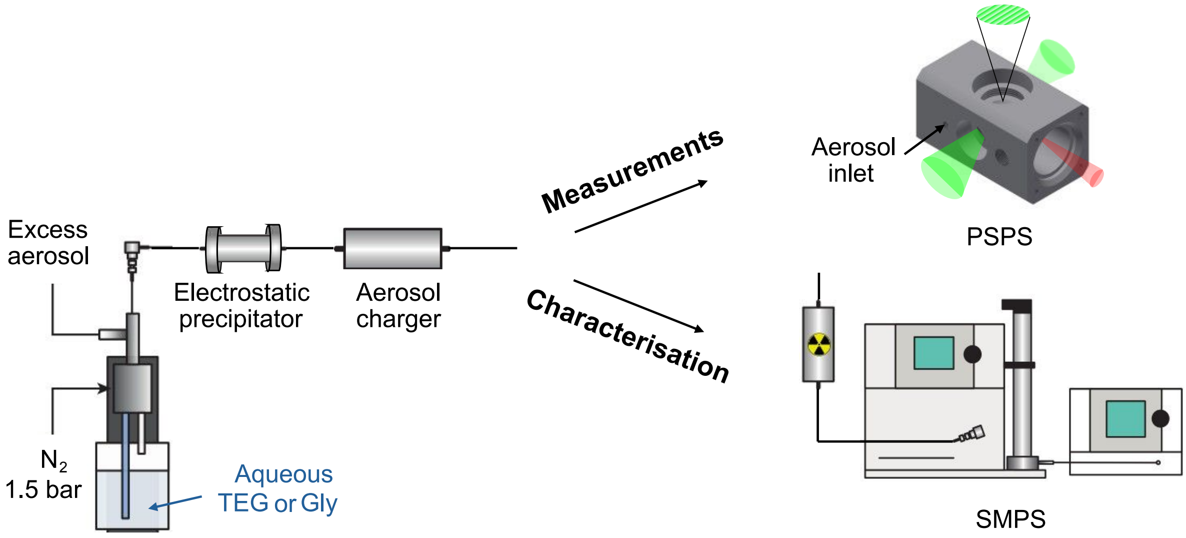

Figure S1: Schematic of the complete setup for droplet generation, charging, and charge state analysis using a Scanning Mobility Particle Sizer (SMPS) spectrometer. Once the electrical charges on the droplets are analysed, we measure them via Photothermal Single-Particle Spectroscopy (PSPS).

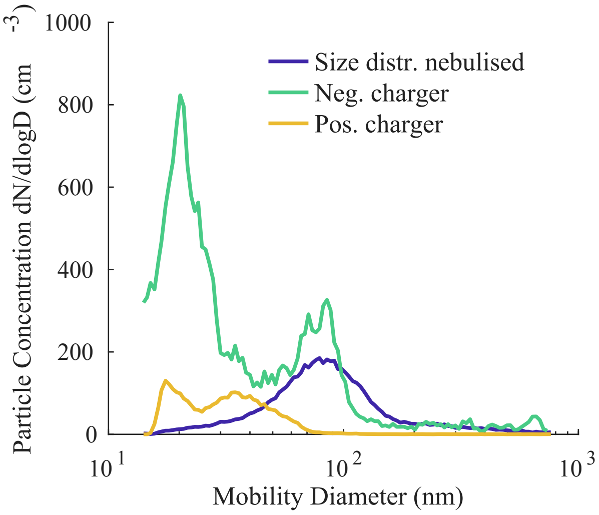

Figure S2: Size distributions of nebulised aqueous Gly droplets depicted as mobility diameters. The blue trace corresponds to the size distribution obtained by equipping the SMPS with the X-ray neutraliser and applying a multiple-charge correction. To obtain the blue distribution, the aerosol ensemble was passed through the turned-off corona-wire charger. The green and the yellow traces indicate the shift in electrical mobilities by charging the droplets with the negative and positive corona-wire chargers, respectively.

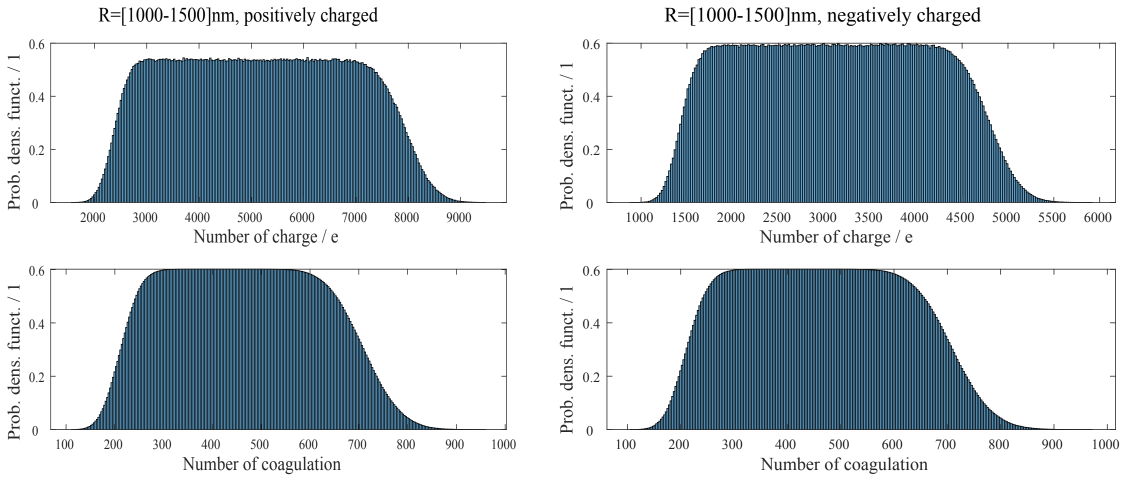

Figure S3: Results of the Monte-Carlo simulation to determine the number of coagulation steps and elementary charges needed to reach the particle size range of 1000 to 1500 nm for positively (left) and negatively charged (right) aqueous Gly droplets.

2. Optical Trapping

The optical setup to trap aqueous TEG and Gly droplets and investigate them with photothermal single particle spectroscopy (PSPS) is described in the main text. A schematic of the full optical setup is provided in Fig. S4, showing the counterpropagating tweezers immobilising a single mixed aqueous-organic droplet in the centre of the photoacoustic cell. The intensity-modulated infrared (IR) laser is focused on the droplet perpendicular to the trapping beams, inducing periodic oscillations in the droplet's thermophysical properties.

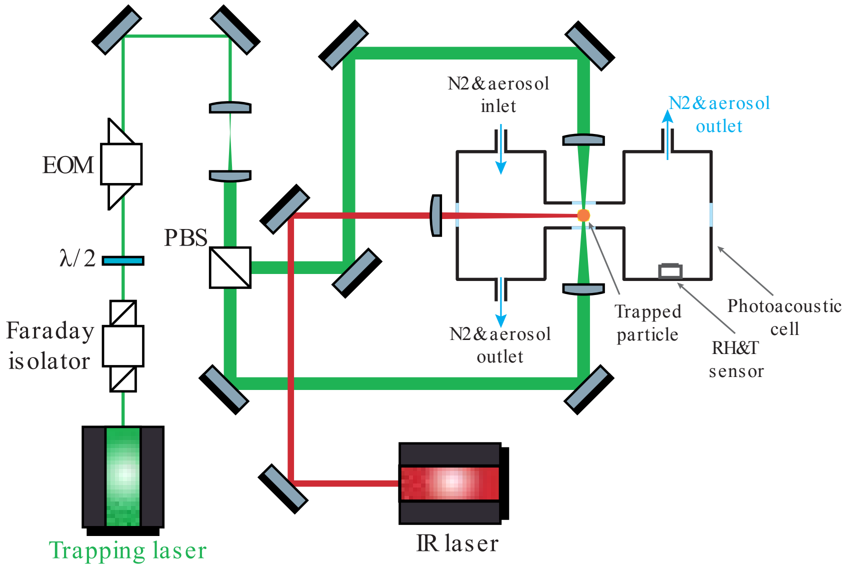

Figure S4: Full optical setup employed for Photothermal single particle spectroscopy. EOM: electro-optical modulator, λ/2: half-wave plate, PBS: polarising beam-splitter cube. The aqueous droplets are immobilised in the centre of the photoacoustic cell. Environmental conditions are controlled by a

humidified N_2 flow and monitored by a relative humidity (RH) and temperature (T) sensor in the cell. The figure is adjusted from Ref.[2]

Table S1: IR laser wavelength, modulation frequency, IR focusing lens, and absorption coefficient *k* at the IR wavelength for the two substances investigated.

Substance	IR wavelength (nm)	Modulation frequency (Hz)	IR Focusing Lens	Absorption coefficient k^a
TEG	9456	3500	*f* = 75 mm, Thorlabs, LA7660-G	0.210
Gly	9680	3525	*f* = 50.8 mm, Edmund Optics, #39-517	0.334

[a] The complex refractive index data of TEG and Gly have been provided by the courtesy of Dr. Tanya Myers from the Pacific Northwest National Laboratory.

3. Model Considerations and Simulation Parameters

The model is described in more depth in our previous publications.[3,4] The general parameters used for the MHM-PA and MMS simulations are listed in Table S2. Specific simulation parameters for the TEG-water and Gly water systems are provided in Table S3 and Table S4, respectively.

Table S2: General parameters for MHM model simulations.

Parameter	Symbol	Value	Reference
Time step	δt	2 ns	-
Layer thickness	d	50 nm	-
Trapping laser wavelength	λ_{trap}	532.15 nm	-
Latent heat	L	$(2440-2453)\ \mathrm{kJ\ kg^{-1}}$ (T dependent)	5
Specific heat capacity (N_2)	$C_{\mathrm{p,N_2}}$	$1040\ \mathrm{J\ kg^{-1}K^{-1}}$	6
Thermal conductivity (N_2)	K	$0.02597\ \mathrm{W\ m^{-1}K^{-1}}$	6
Thermal accommodation coefficient	α_{T}	0.97	4,7,8
Objective opening angle	ϕ_{TTAOS}	24.49°	-

Table S3: Parameters for MHM-PA and MMS simulations for aqueous TEG droplets.

Parameter	Symbol	Value	Reference
Excitation laser wavelength	λ_{IR}	9456 nm	-
Diffusion coefficient	D_{w}	$(10-30)\cdot 10^{-11}\ \mathrm{m^2s^{-1}}$ (RH and T dependent)	9,10
Thermal diffusivity	D_{h}	$(7.1-7.2)\cdot 10^{-8}\ \mathrm{m^2s^{-1}}$ (RH and T dependent)	9
Specific heat capacity (TEG)	C_{p}	$(2140-4181)\ \mathrm{J\ kg^{-1}K^{-1}}$	6,9

		(RH and T dependent)	
Density of aqueous TEG	ρ	$(0.998-1.124)\ \mathrm{g\ cm^{-3}}$ (RH and T dependent)	[6,9]
Refractive index TEG at λ_{IR}	$n_{IR}^{\mathrm{TEG}}+ik_{IR}^{\mathrm{TEG}}$	$(1.624-1.627)+i\ 0.210$(T dependent)	PNNL[a]
Refractive index TEG at λ_{trap}	$n_{trap}^{TEG}+ik_{trap}^{TEG}$	$(1.469-1.472)+i\ 2.48\cdot10^{-8}$ (T dependent)	[9,11], PNNL[a]
Refractive index water at λ_{IR}	$n_{IR}^{\mathrm{H_2O}}+ik_{IR}^{\mathrm{H_2O}}$	$1.2448+i\ 0.0439$	[12]
Refractive index water at λ_{trap}	$n_{trap}^{\mathrm{H_2O}}+ik_{trap}^{\mathrm{H_2O}}$	$(1.335-1.336)+i\ 1.499\cdot10^{-9}$ (T dependent)	[13]
Partial vapour pressure water	P_{w}	$(250-4040)\ \mathrm{Pa}$ (RH and T dependent)	[6,14]

[a] The complex refractive index data of aqueous Gly was provided by the courtesy of Dr. Tanya Myers from the Pacific Northwest National Laboratory.

Table S4: Parameters for MHM-PA and MMS simulations for aqueous Gly droplets.

Parameter	Symbol	Value	Reference
Excitation laser wavelength	λ_{IR}	9680 nm	-
Diffusion coefficient	D_{w}	$(9\cdot10^{-12}-1\cdot10^{-9})\ \mathrm{m^2s^{-1}}$ (RH and T dependent)	[15,16]
Thermal conductivity	K_{Gly}	$0.2842-0.6015\ \mathrm{W\ m^{-1}K^{-1}}$ (RH and T dependent)	[17]
Specific heat capacity (Gly)	C_{p}	$903-4181\ \mathrm{J\ kg^{-1}K^{-1}}$ (RH and T dependent)	[6,18]
Density of aqueous TEG	ρ	$0.998-1.261\ \mathrm{g\ cm^{-3}}$ (RH and T dependent)	[19]
Refractive index Gly at λ_{IR}	$n_{IR}^{\mathrm{Gly}}+ik_{IR}^{\mathrm{Gly}}$	$(1.6368-1.6334)+i\ 0.3337$(T dependent)	PNNL[a]
Refractive index Gly at λ_{trap}	n_{trap}^{Gly}	$1.4764-1.4703$(T dependent)	[20], PNNL[a]
Mass fraction Gly	m_{Gly}	$0.20-0.99$ (RH and T dependent) [b]	-

[a] The complex refractive index data of aqueous TEG was provided by the courtesy of Dr. Tanya Myers from the Pacific Northwest National Laboratory. [b] Gly concentrations (in mass fraction and mole fraction) were obtained from our own Raman measurements of aq. Gly droplets at temperatures between 21 and 33 °C and RH values ranging from 2 to 97%. These measurements are included in the data repository of this work.

4. Glycerol MHM-PA Simulation

While a careful evaluation of the MMS traces for aqueous TEG droplets yielded absolute values for the mass accommodation coefficient α_M, we could not extend this analysis to Gly droplets, particularly at low relative humidities (RH).

The most plausible explanation for the failure of the analysis using MHM-PA simulations[3,4] is that the system deviates too far from equilibrium under these conditions. For example, at 18% RH, ΔT reaches approximately 13 K for droplets with an average radius of $\bar{r}$ = 1.5 µm (see Fig. S5), and 7.5 K for $\bar{r}$ = 1.0 µm. Such strong departures from equilibrium can substantially distort the retrieval of α_M, leading to unreliable results.[21]

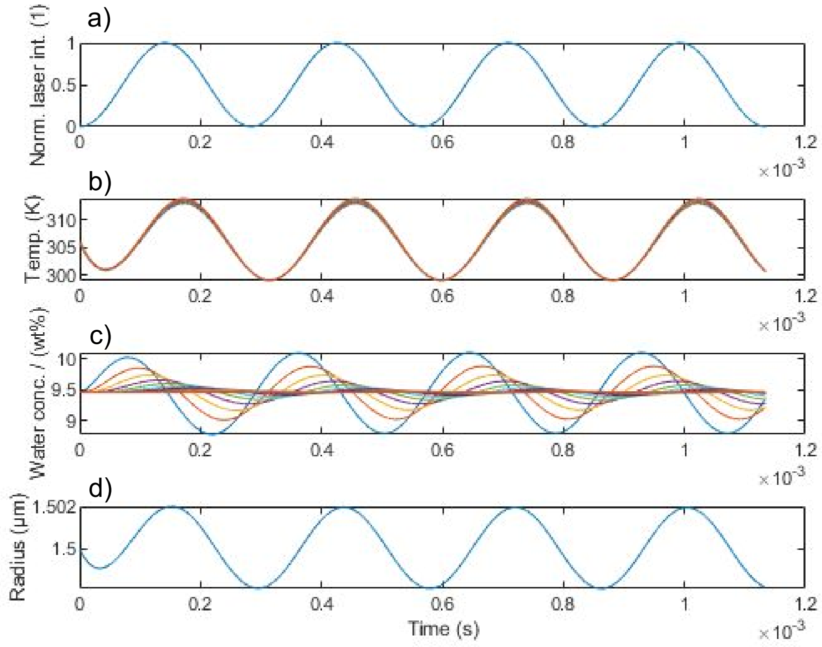

Figure S5: Simulated evolution of the a) normalised laser intensity, b) temperature in the different layers, c) water concentration in the different layers, and d) the droplet radius as a function of time during four photothermal cycles at a modulation frequency of 3525 Hz for an aqueous Gly droplet with $\bar{r} = 1.5$ µm at 18% RH.

An important consideration for the MHM-PA model is the assumption of homogeneous light distribution within the droplet. Fig. S6 shows an ADDA simulation of the internal light field in a Gly droplet under dry conditions (0% RH), which corresponds to the strongest IR absorption scenario. Despite these extreme conditions the simulation supports the validity of the homogeneous light distribution assumption. However, even with a uniform internal light field, the magnitude of absorption may still be excessive, potentially driving the system far from equilibrium and contributing to the deviations discussed above.

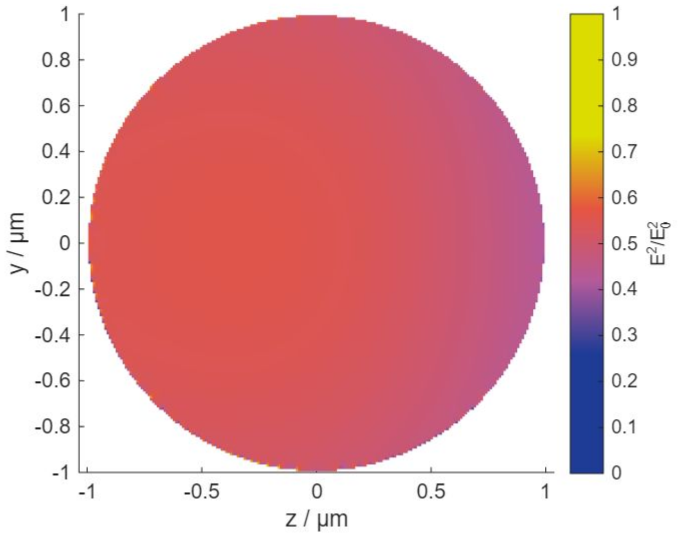

Figure S6: ADDA simulation of the infrared light intensity inside a Gly droplet for an IR wavelength of 9.68 µm, *r* = 1.0 µm, *n* = 1.63 + *i*0.336 (pure Gly). The laser propagation direction is along the z-direction, with a Gaussian beam width $2\omega_0 = 105\ \mu\text{m}$. The colour bar indicates the relative internal light distribution E^2 compared to the incident light intensity $E_0{}^2$.

5. <u>Mass accommodation coefficient of water on TEG</u>

As mentioned in the main manuscript in Section 3.2 and shown in Fig. 5, we do not observe systematic differences in the retrieved α_M values between uncharged, positively charged, and negatively charged TEG droplets. To facilitate direct visual comparison, α_M data from Fig. 5 are overlaid in Fig. S7, grouped into two temperature ranges: a) 21-23 °C and b) 23-26 °C.

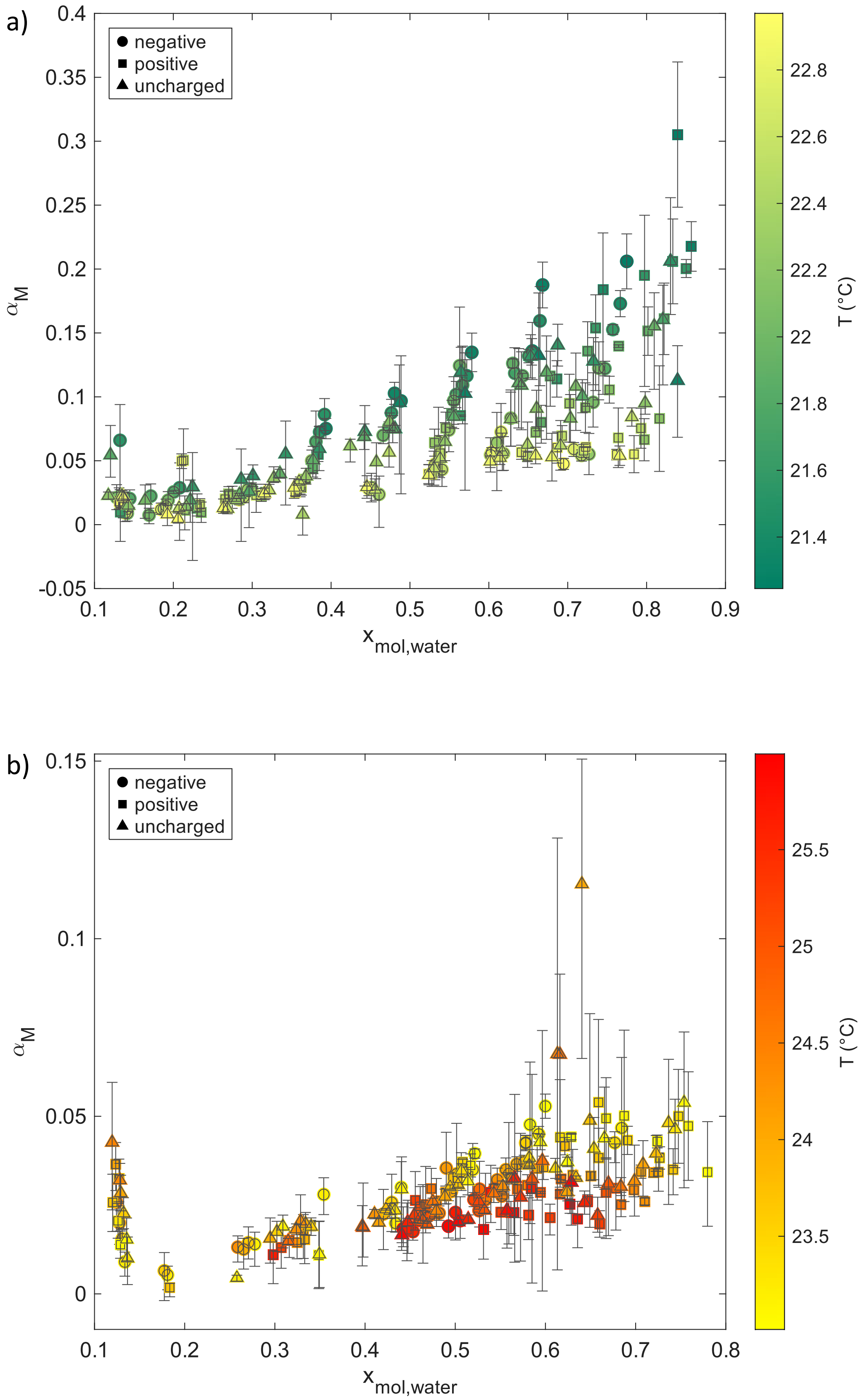

Figure S7: α_M data from Fig. 5 overlaid and grouped into two droplet temperature ranges a) from 21 to 23 °C and b) 23 to 26 °C. Error bars represent the average fitting uncertainty in α_M. Round, square,

and triangular markers represent negatively charged, positively charged, and uncharged TEG droplets, respectively.

6. Charge distribution at the surface and interaction with water molecules

We describe the interactions between an incoming water molecule with the charged droplet surface by the angle-averaged charge-dipole interaction energy given by Eq. (8) in the main text. For a spherical droplet with radius r = 1000 nm and 1500 elementary charges, the area per charge is 0.0084 μm^2. Assuming a hexagonal arrangement of the charges at the droplet surface, to maximise their mutual distances, we estimate an average inter-charge spacing of approximately 88 nm. In a Monte-Carlo simulation with 10^6 random points to sample the positions within the domain, we find that the average distance from a randomly placed point to the nearest charge is ~33 nm. This corresponds to the average distance of an incoming dipole (water molecule) to a charge.

This model was further employed to estimate charge-dipole interaction energies. Similarly, we constructed a numerical model to estimate the dipole-dipole interaction energies. In both cases, the energies were integrated over a representative surface area to include interactions with neighbouring charges and dipoles, respectively, and the incoming water molecule was incorporated into the surface layer. Additionally, we included a minimum distance – set as the typical molecular distance in water – between incoming water molecule and charge or dipole to prevent the energy formulas (Eqs. (8) and (9) in main) from diverging to infinity at very short distances.

References

(1) Parmentier, E. A.; Corral Arroyo, P.; Gruseck, R.; Ban, L.; David, G.; Signorell, R. Charge Effects on the Photodegradation of Single Optically Trapped Oleic Acid Aerosol Droplets. *Journal of Physical Chemistry A* **2022**, *126* (27), 4456–4464. https://doi.org/10.1021/acs.jpca.2c01370.

(2) Roy, S.; Diveky, M. E.; Signorell, R. Mass Accommodation Coefficients of Water on Organics from Complementary Photoacoustic and Light Scattering Measurements on Laser-Trapped Droplets. *Journal of Physical Chemistry C* **2020**, *124* (4), 2481–2489. https://doi.org/10.1021/acs.jpcc.9b09934.

(3) Corral Arroyo, P.; Gleichweit, M. J.; Diveky, M. E.; Signorell, R. A Multilayer Heat and Mass Transfer Model for Photoacoustics of Aerosol Particles (MHM-PA). *Aerosol Science and Technology* **2023**, *57* (8), 742–757. https://doi.org/10.1080/02786826.2023.2206450.

(4) Gleichweit, M. J.; Azizbaig Mohajer, M.; Borgeaud dit Avocat, D. P.; Divéky, M. E.; David, G.; Signorell, R. Unexpected Concentration Dependence of the Mass Accommodation Coefficient of Water on Aqueous Triethylene Glycol Droplets. *Physical Chemistry Chemical Physics* **2024**, *26* (22), 16296–16308. https://doi.org/10.1039/d4cp00966e.

(5) Rogers, R. R.; Yau, M. K. *A Short Course in Cloud Physics*; Elsevier, 1996.

(6) Rumble, J. *Handbook of Chemistry and Physics*, 99th ed.; CRC Press, Taylor & Francis Group, 2018.

(7) Winkler, P. M.; Vrtala, A.; Rudolf, R.; Wagner, P. E.; Riipinen, I.; Vesala, T.; Lehtinen, K. E. J.; Viisanen, Y.; Kulmala, M. Condensation of Water Vapor: Experimental Determination of Mass and Thermal Accommodation Coefficients. *Journal of Geophysical Research Atmospheres* **2006**, *111* (D19), 1-12. https://doi.org/10.1029/2006JD007194.

(8) Murphy, D. M. The Effect of Water Evaporation on Photoacoustic Signals in Transition and Molecular Flow. *Aerosol Science and Technology* **2009**, *43* (4), 356–363. https://doi.org/10.1080/02786820802657392.

(9) The Dow Chemical Company. *Tetraethylene Glycol*; 2007.

(10) Paduano, L.; Sartorio, R.; Dïerrico, G.; Vitagliano, V. Mutual Diffusion in Aqueous Solution of Ethylene Glycol Oligomers at 25 C. *Journal of the Chemical Society* **1998**, *94* (17), 2571-2576.

(11) Otanicar, T. P.; Phelan, P. E.; Golden, J. S. Optical Properties of Liquids for Direct Absorption Solar Thermal Energy Systems. *Solar Energy* **2009**, *83* (7), 969–977. https://doi.org/10.1016/j.solener.2008.12.009.

(12) Hale, G. M.; Querry, M. R. Optical Constants of Water in the 200-nm to 200-μm Wavelength Region. *Optica* **1973** 12 (3), 555-563. https://doi.org/10.1364/OA_License_v1#VOR.

(13) Bashkatov, A. N.; Genina, E. A. Water Refractive Index in Dependence on Temperature and Wavelength: A Simple Approximation; SPIE 2003; p. 393-395.

(14) Lange, N. A. *Langés Handbook of Chemistry*, 17th ed.; Speight, J. G., Ed.; McGraw-Hill Education: New York, 2017.

(15) Chen, Y.-M.; Pearlstein, A. J. Viscosity-Temperature Correlation for Glycerol-Water Solutions. *Ind. Eng. Chem. Res.* **1987**, *26* (8), 1670–1672.

(16) D'Errico, G.; Ortona, O.; Capuano, F.; Vitagliano, V. Diffusion Coefficients for the Binary System Glycerol + Water at 25 °C. a Velocity Correlation Study. *J Chem Eng Data* **2004**, *49* (6), 1665–1670. https://doi.org/10.1021/je049917u.

(17) Bates, O. K. Binary Mixtures of Water and Glycerol. *Ind Eng Chem* **1936**, *28* (4), 494–498.

(18) Shah, B. N.; Chinte, U.; Tomanicek, S. J.; Leif Hanson, B.; Schall, C. A. Flash Cooling Protein Crystals: Estimate of Cryoprotectant Concentration Using Thermal Properties. *Cryst Growth Des* **2011**, *11* (5), 1493–1501. https://doi.org/10.1021/cg1013939.

(19) Volk, A.; Kähler, C. J. Density Model for Aqueous Glycerol Solutions. *Experiments in Fluids* **2018**, *59* (5), 75. https://doi.org/10.1007/s00348-018-2527-y.

(20) Chabouni, Y.; Amireche, F. Some Physicochemical Properties of Binary Mixtures of Glycerol with Butanol Isomers in the Temperature Range 293.15-318.15 K and Ambient Pressure. *J Chem Eng Data* **2020**, *65* (4), 1679–1694. https://doi.org/10.1021/acs.jced.9b01052.

(21) Zientara, M.; Jakubczyk, D.; Kolwas, K.; Kolwas, M. Temperature Dependence of the Evaporation Coefficient of Water in Air and Nitrogen under Atmospheric Pressure: Study in Water Droplets. *Journal of Physical Chemistry A* **2008**, *112* (23), 5152–5158. https://doi.org/10.1021/jp7114324.
